# Building a Simple Platform for Tailoring Peptide Surface Chemistry to Enhance Cellular Uptake of Polymer-Coated Gold Nanoparticles

**DOI:** 10.1021/acsomega.5c10366

**Published:** 2026-02-25

**Authors:** Ilya Kotelnikov, Gabriela Borba Mondo, Caroline Arana da Silva Ribeiro, Maria Mercedes Rolon Sosa, Wendel Andrade Alves, Bruno Lemos Batista, Ognen Pop-Georgievski, Vladimir Proks, Cristiano Giacomelli, Fernando Carlos Giacomelli

**Affiliations:** a Institute of Macromolecular Chemistry, 86879 Czech Academy of Sciences, Prague 162 00, Czech Republic; b Centro de Ciências Naturais e Humanas, 74362 Universidade Federal do ABC, Santo André 09280-560, Brazil; c Departamento de Química, Centro de Ciências Naturais e Exatas, Universidade Federal de Santa Maria, Santa Maria 97105-900, Brazil

## Abstract

Nanotherapeutic delivery requires particulate systems composed of only a few components yet featuring multiple capabilities to reach the clinic. In this study, we built a platform based on selective reactions (solid-phase peptide synthesis, peptide coupling chemistry, and click chemistry) to enhance cellular uptake of gold nanoparticles (AuNPs) by anchoring cell-penetrating peptides (CPPs) at the outermost layer of the constructs. Model amphipathic peptide (MAP), trans-acting activator of transcription (TAT), hexarginine (R_6_), and arginine monomer (Arg) were attached to the surface of AuNPs synthesized directly by using branched polyethylenimine (BPEI) polyelectrolyte. From a different perspective, a water-soluble CGSWQWRR sequence capable of promoting the reduction of auric species and steric stabilization of gold colloids was also synthesized. The structure and dynamics of particles were characterized using imaging, scattering, and spectroscopy techniques, and their biological performance was evaluated by assessing cell viability and cellular uptake. The nanoconstructs were noncytotoxic up to 1.0 ppm in the case of CPP–BPEI@AuNPs (5.2 nm metallic core) or up to 10 ppm for CGSWQWRR@AuNPs (30 nm metallic core). Those featuring CPPs at the surface were internalized faster and to a higher extent (∼40% in 4 h) compared to the precursor (∼20% in 4 h). The highest cellular uptake was found for CGSWQWRR@AuNPs (∼75% in 4 h), which mediated the membrane-wrapping process more effectively and was prepared by the easiest protocol (one-pot, two-reactant, no workup reaction in aqueous media). The findings of this study simplify nanoparticle manufacturing, thereby reducing the gap between chemical synthesis and clinical applications.

## Graphical abstract

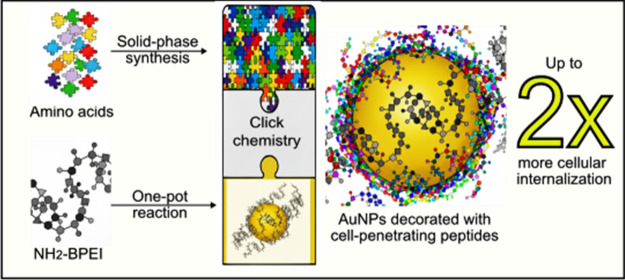

## Introduction

The success of nanotheranostics as a holistic, personalized and patient-centered medical strategy is intrinsically linked to the precise modulation of nanoparticle-cell interactions.^1^ In turn, this implies that the key factor in the process of designing and manufacturing such systems is the surface chemistry, which should target the cellular level with precision.^2^ This, however, is no easy task due to the inherent complexity of biological media. Despite significant advances in the field, the applications of nanoobjects in medicine still face several limitations, challenges, and barriers, including rapid clearance, off-target localization, degradation, and nonspecific interactions with serum proteins, thus leading to protein fouling.^3^

One of the most effective ways to overcome these issues altogether is engineering particles that mimic living cells.^4^ On the one hand, this is the reason why proteins, peptides,^5–8^ and small molecule sugars^9^ have proven superior capability for targeting cellular environments compared to synthetic polymers, which, on the other hand, have demonstrated good performance in applications involving the synthesis and steric stabilization of colloids, but limited cell specificity. Combining both features into one robust, environmentally friendly, cost-effective, and easy-to-scale-up platform is of high industrial interest for nanotherapeutic delivery, especially when the synthetic route offers facile tailoring via approaches that utilize interlocking building blocks. It is also necessary to consider that nanoconstructs must be simple and composed of only a few components to reach the clinic,^6,10,11^ otherwise they remain limited to proof-of-concept endeavors.

In this study, we utilize typical amine chemistry as a pathway to meet the criteria as mentioned above while building a robust platform for tailoring the peptide surface chemistry of polymer-coated gold nanoparticles (AuNPs). The applications of AuNPs in medicine are of a broad spectrum, and translocation of nanoobjects across the cellular lipid membrane is required in many cases. Peptides can be used to functionalize AuNPs, thus enabling selective binding to receptors on target cells and facilitating drug delivery with enhanced cellular uptake and reduced off-target effects.^12^ Peptide coatings can also improve the responsiveness of gold assemblies used in radiation-based therapies,^13^ while also imparting capability of targeting specific sites in molecular imaging applications.^14^ Within this context, the aim of this study was to establish a robust platform to enhance cellular uptake by anchoring specific peptides onto the surface of AuNPs. The gap it fills is the lack of a modular strategy for peptide functionalization of nanoconstructs using a method that allows for the choice of building blocks from a library while keeping the chemistry constant; it is modular because any peptide can be anchored at the surface of gold colloids.

Bridging such a gap was envisioned via a combination of simple, yet efficient, techniques. Here, a simple approach is meant using an amino-functionalized polyelectrolyte known to successfully mediate the formation of stable gold colloids in one-pot, two-reactant, no workup reactions in aqueous media,^15,16^ followed by conversion of unreacted amino groups into alkynes for rapid click-chemistry type attachment of virtually any peptide of interest at the outermost surface of the hybrid nanoparticle.

The oxidation chemical groups from macromolecules capable of mediating the reduction of gold species and, accordingly, the direct synthesis of AuNPs, generate reactive species as primary products. Such species eventually prompt an oxidative polymerization process that results in the formation of a polymeric coating consisting of several chains arranged in a star-like structure on the nanoparticle surface, as discovered by Newman and Blanchard^17^ while studying the formation of AuNPs using low-molecular-weight amine reducing agents. From a reaction stoichiometry point of view, a large excess (3-fold or more) of reducing agent has been normally used in these reactions as compared to the oxidizing agent. The direct implication of this experimental procedure is that a considerable amount of unreacted electron-donor groups remains available for functionalization of the nanoparticle surface after it has been synthesized. This is the case with branched polyethylenimines (BPEI), a polyelectrolyte that acts simultaneously as a reducing and stabilizing agent without the aid of any other external agent. It is interesting to note that BPEI could, in principle, be functionalized before AuNPs synthesis.^18^ Such a pathway, however, is not ideal for peptide functionalization because this latter class of molecules may also display all the attributes necessary to effectively mediate the reduction of gold species, thereby being subjected to potential conversion into unknown products that may not feature cell-targeting or cell-penetrating abilities.

Keeping the overall synthetic route simple and cost-effective, we elected to use a typical peptide coupling methodology (also applied for solid-phase peptide synthesis) to react amine groups at the surface of AuNPs with 4-pentynoic acid in the presence of an oxime as an additive.^19^ An amide/peptide bond is formed, with alkyne groups exposed at the particle’s surface and, therefore, available for further biomimetic modifications with the azido-functionalized peptides by Cu­(I)-catalyzed azide–alkyne cycloaddition (click reaction). We believe this is a feature of the platform built herein, as it is founded on well-known, highly selective reactions: solid-phase peptide synthesis, peptide coupling chemistry, and click chemistry.

The short-sequenced and positively charged peptides selected for this study belong to the so-called class of cell-penetrating peptides (CPPs), which are known to translocate across lipid membranes without compromising the structural integrity of cells, thus enabling transport of extracellular materials (substances and particles) into cells.^5,7,20,21^ They are hexarginine (R_6_), trans-acting activator of transcription (TAT), the model amphipathic peptide (MAP), and two other sequences synthesized with inspiration by a cationic peptide derived from bovine LfcinB; R_6_ comprises six arginine residues of RRRRRR sequence; TAT peptide is derived from the human immuno-deficiency virus (HIV) and consists of the sequence RKKRRQRRR with six arginine residues along with two lysine residues and one glutamine residue;^21^ MAP is the sequence KLALKLALKALKAALKLA with lysine, leucine and alanine residues;^5^ the other peptide was based on the WQWRR sequence with arginine, tryptophan, glutamine, but with an extension with cysteine, glycine and serine to yield CGSGWQWRR (hereinafter referred to as CGS as short form) to improve solubility in water and capability to mediate the reduction of auric species.

## Experimental Section

### Materials and Chemicals

Chloroauric acid trihydrate, branched polyethylenimine (BPEI, *M_w_* = 25,000 g·mol^–1^, *M_w_*/*M*_*n*_ = 2.5), amino acids for manual peptide synthesis, Fmoc-Arg-Wang resin, azidoacetic acid, 4-pentynoic acid, ACS grade DMF, acetic acid, sodium borohydride, and sodium borate were of the highest purity available from Sigma-Aldrich and used as received. Amino acids Fmoc-Lys­(Boc)–OH, *Fmoc-Ala-OH, Fmoc-Lys­(Boc)–OH, Fmoc-Leu-OH, Fmoc-Gln­(Trt)–OH, Fmoc-Arg­(Pbf)–OH,* ethyl­(hydroxyimino)­cyanoacetate (Oxyma Pure), diisopropylcarbodiimide (DIC), piperidine, peptide grade DMF, triisopropylsilane (TIS), trifluoroacetic acid (TFA), and thioanisole for peptide synthesis in automatic synthesizer were purchased from Iris Biotech GmbH. TentaGel R RAM resin (amine content 0.19 mmol·g^–1^) was purchased from RAPP-polymers. DMEM/F12 GlutaMAX supplement, Dulbecco’s Modified Eagle Medium, and 3-(4,5-dimethylthiazol-2yl)-2,5-diphenyl-tetrazolium bromide (MTT) were acquired from Thermo Fisher Scientific. Ultrapure water was obtained with a Milli-Q Plus System (Millipore Corporation).

### Solid-Phase Peptide Synthesis

Azide-terminated peptides were synthesized by the standard solid-phase Fmoc/tBu protocol on a TentaGel R RAM high swelling resin, as previously described elsewhere.^22^ Briefly, an automatic CEM Liberty Blue microwave peptide synthesizer was used to carry out the reactions in DMF, using default DIC/Oxyma Pure coupling and piperidine deprotection cycles. Amino acid coupling and Fmoc deprotection occurred at 90 °C under microwave heating - a piperidine 20% solution treatment induced Fmoc release. Azidocetoyl-G_3_R_6_-NH_2_ (R6) and azidocetoyl-GGGRKKRRQRRR-NH_2_ (TAT) were cleaved from the resin in a TFA/thioanisole/water/TIS 91:3:5:1 *v*/*v*/*v*/*v* mixture during 3 h and then purified by precipitation in diethyl ether followed by freeze-drying. Bruker UltrafleXtreme MALDI-TOF mass spectrometer runs confirmed the identity of reaction products. Azidocetoyl-GGGKLALKLALKALKAALKLA-NH_2_ (MAP) was cleaved from the resin in a TFA/DCM 1:1 *v*/*v* mixture containing 1% TIS after stirring for 1 h. Subsequently, the resin was filtered off, and the reaction mixture was stirred for an additional 1 h, then evaporated and precipitated in diethyl ether. NH_2_–CGSGWQWRR–OH peptide was synthesized via a manual approach by applying the standard Fmoc/tBu protocol on Fmoc­(Arg)-Wang resin on glass frits and was cleaved using a mixture of TFA/thioanisole/water/DTT/TIS at a 90:3:5:1:1 *v*/*v*/*v*/*v* ratio. After purification, the expected reaction products were confirmed by LC-MS.

### Synthesis of Peptide-Coated AuNPs

#### Step 1: Synthesis of BPEI-Capped Gold Colloids

Organic solutions of HAuCl_4_ (1.0 mg·mL^–1^; 1.5 mL) and BPEI (1.0 mg·mL^–1^; 1.5 mL) in pure and amine-free DMF were mixed in a reaction vessel (total reaction volume of 3.0 mL) that was immediately immersed in an oil bath at 50 °C. The reaction mixture was stirred for 10 h, then left at room temperature overnight. The formation of the gold nanoparticles was evidenced by the typical color originating from their characteristic localized surface plasmon resonance (SPR). The obtained nanoparticles in DMF were used in the next step for alkyne functionalization. For biological assays, particles were transferred to water to a final volume of 6.0 mL. This colloidal sample is referred to as NH_2_–BPEI@AuNPs throughout the manuscript.

#### Step 2a: Synthesis of Alkyne-Functionalized Gold Colloids

A mixture of 10.0 mg of 4-pentynoic acid activated with 28.0 mg of Oxyma Pure and 15.0 μL of DIC dissolved in 1.0 mL of DMF was poured into 3.0 mL of NH_2_–BPEI@AuNPs in DMF previously prepared in step 1. Then, the reaction mixture was stirred for 4 h before being diluted with 60 mL of water. The resulting suspension was concentrated and washed three times with water using Amicon Ultra Centrifugal Filters with 100 kDa MWCO. This colloidal sample is referred to as HC≡C–BPEI@AuNPs throughout the manuscript.

#### Step 3a: Synthesis of Peptide-Decorated AuNPs

Initially, stock solutions of peptides were prepared by dissolving 2.5 μmol of each sample (3 mg of R_6_, 4 mg of TAT or 5 mg of MAP) in 1.0 mL of water, followed by 10-fold dilution. Then, a solution of HC≡C–BPEI@AuNPs was concentrated to approximately 1 mL. To the resulting solution from this procedure, 100 μL of a 20 mg·mL^–1^ sodium ascorbate solution and 20 μL of the peptide solution were added in sequence. Next, the mixture was purged with a gentle nitrogen gas flow for 15 min before the addition of 40 μL of 0.05 mol·L^–1^ CuSO_4_, followed by further purging with nitrogen gas for additional 30 min. The particles were washed using Amicon Ultra Centrifugal Filters as previously described. These colloidal samples are referred to as R_6_–BPEI@AuNPs, TAT–BPEI@AuNPs, and MAP–BPEI@AuNPs throughout the manuscript, or more generically as CPP–BPEI@AuNPs.

#### Step 2b: Synthesis of AuNPs Decorated with Arginine Having Protected Guanidino Group

Reaction mixtures containing varying amounts of Fmoc-Arg­(NO_2_)–OH (1, 3, and 8 mg), activated by Oxyma Pure (1, 2, and 5 mg) and DIC (0.5, 1, and 3 μL), were added to separate vessels containing 3.0 mL of NH_2_–BPEI@AuNPs in DMF previously prepared in step 1. Then, the reaction mixtures were stirred for 4 h before being diluted with 60 mL of water. The resulting suspensions were washed following the protocol already described in step 2a, as are denoted Arg­(NO_2_)–OH–BPEI@AuNPs.

#### Step 3b: Synthesis of Arginine-Decorated AuNPs

Nitro groups in Arg­(NO_2_)–OH–BPEI@AuNPs were released by sodium borohydride treatment. Ten-mL aliquot of DMF/MeOH/AcOH (9:9:2 *v*/*v*/*v*) solution containing 20 mg of NaBH_4_ was added to Arg­(NO_2_)–OH–BPEI@AuNPs, and the mixture was kept under stirring for 30 min. After this elapsed time, the reaction mixture was diluted with 150 mL of water, washed, and concentrated as described above. This sample is referred to as Arg–BPEI@AuNPs throughout the manuscript.

#### Direct One-Pot Synthesis of CGS@AuNPs

The synthesis of CGS@AuNPs is an exception to the multistep synthetic route. These particles were prepared directly in aqueous media by reacting CGSWQWRR (full sequence) peptide (1.0 mg·mL^–1^; 1.5 mL) with HAuCl_4_·3H_2_O (1.0 mg·mL^–1^; 1.5 mL). In this case, the peptide acts simultaneously as a reducing and stabilizing agent, according to the reaction pathways already reported.^15,16^ The chemical reactions were carried out at room temperature until the UV–vis absorption remained nearly constant over time (∼ 4 h).

### Characterization of the Gold Colloids

#### Dynamic Light Scattering (DLS)

The autocorrelation functions were acquired at 25 °C using an ALV/CGS-3 platform-based goniometer system (ALV GmbH) equipped with a polarized HeNe laser (22 mW) at a wavelength of 633 nm, an ALV 7004 digital correlator, and a pair of APD-based single-photon detectors. The resulting relaxation time distributions were obtained using the CONTIN algorithm and further converted into hydrodynamic radius (*R*_H_) distributions by using the Stokes–Einstein equation:

$$R_{H}=\frac{k_{B}Tq^{2}}{6\pi \eta }\tau$$ 1

where *k*_*B*_ is the Boltzmann constant, *T* is the absolute temperature, *q* is the scattering vector, η is the solvent viscosity, and τ is the mean relaxation time. The autocorrelation functions (*g*_1_(*t*)) were also analyzed using the Cumulant method (second-order cumulant).^23^

$$\ln\;g_{1}\left(t\right)=\ln\;C-\Gamma t+\frac{\mu_{2}}{2}t^{2}$$ 2

where *C* is the amplitude of the autocorrelation function, Γ is the relaxation frequency (τ^–1^), and the parameter *μ*_*2*_ is known as the second-order cumulant. This approach enabled the determination of polydispersity indexes (PDI = μ_2_/Γ^2^) for monomodal distributions of sizes.

#### Electrophoretic Light Scattering (ELS)

The electrophoretic mobility of the prepared particles was measured using a Malvern Zetasizer Nano-ZS apparatus. The ζ-potential (zeta potential) values were derived from electrophoretic mobility (*U*_E_) data using the Henry equation and assuming the Smoluchowski approximation for aqueous samples (f­(κa) = 1.5).^24,25^

$$U_{E}=\frac{2\varepsilon \zeta }{3\eta }f\left(\kappa a\right)$$ 3

where ε is the dielectric constant of the medium and η its viscosity.

#### Transmission Electron Microscopy (TEM)

The peptide-coated AuNPs were imaged using a Tecnai G2 Spirit Twin 120 kV (FEI, Czech Republic) microscope. Two microliters of each sample (c = 10 ppm) were deposited onto a copper TEM grid (400 mesh) coated with a thin carbon film. The excess of samples was removed by touching the bottom of the grid with filtering paper. The grids were left to dry completely at room temperature before imaging. Particle size analysis was performed by manual inspection and measurement of TEM micrographs. A minimum of 300 particles from different regions of the grid were probed and only well-resolved, nonoverlapping particles were considered. The particle size distributions were obtained by measuring the diameter, and results are reported as mean ± standard deviation.

#### X-ray Photoelectron Spectroscopy (XPS)

XPS measurements were performed using a K-Alpha^+^ spectrometer (ThermoFisher Scientific, East Grinstead, UK). Gold nanoparticles featuring different surface coatings that were representative of each step undertaken along the synthetic route according to Scheme 1 (step 1: NH_2_–BPEI@AuNPs, step 2a: HC≡C–BPEI@AuNPs, and step 3a: TAT–BPEI@AuNPs) were spread on freshly cleaned silicon wafers. The resulting films were analyzed using a microfocused (spot radius of 400 μm), monochromated Al Kα X-ray source at an angle of incidence of 30° (measured from the surface) and an emission angle normal to the surface. The kinetic energy of the electrons was measured using a 180° hemispherical energy analyzer operated in constant analyzer energy (CAE) mode at 200 and 50 eV pass energies for the survey and high-resolution spectra, respectively. Survey and high-resolution spectra were measured. The high-resolution spectra were measured in the region of Au 4f, Cl 2p, C 1s, N 1s, and O 1s. Spectral resolutions of 0.1 and 1.0 eV were used for the high-resolution and survey spectra, respectively. All reported XPS spectra are averages of the 10 individual measurements referenced to the C 1s peak of hydrocarbons at 285.0 eV. Data acquisition and processing were performed using Thermo Advantage software. The XPS spectra were fitted with Voigt profiles obtained by convolving Lorentzian and Gaussian functions. The analyzer transmission function, Scofield sensitivity factors, and effective attenuation lengths (EALs) for photoelectrons were applied for quantification. EALs were calculated using the standard TPP-2 M formalism. The BE scale was controlled by the well-known position of the photoelectron C–C and C–H, C–O, and C­(=O)-O C 1s peaks of polyethylene terephthalate and Cu 2p, Ag 3d, and Au 4f peaks of metallic Cu, Ag, and Au, respectively. The BE uncertainty of the reported measurements and analysis did not exceed ± 0.2 eV. The quantitative analysis and reported values are averages of 5 measurements on independently prepared samples.

**Scheme 1:**
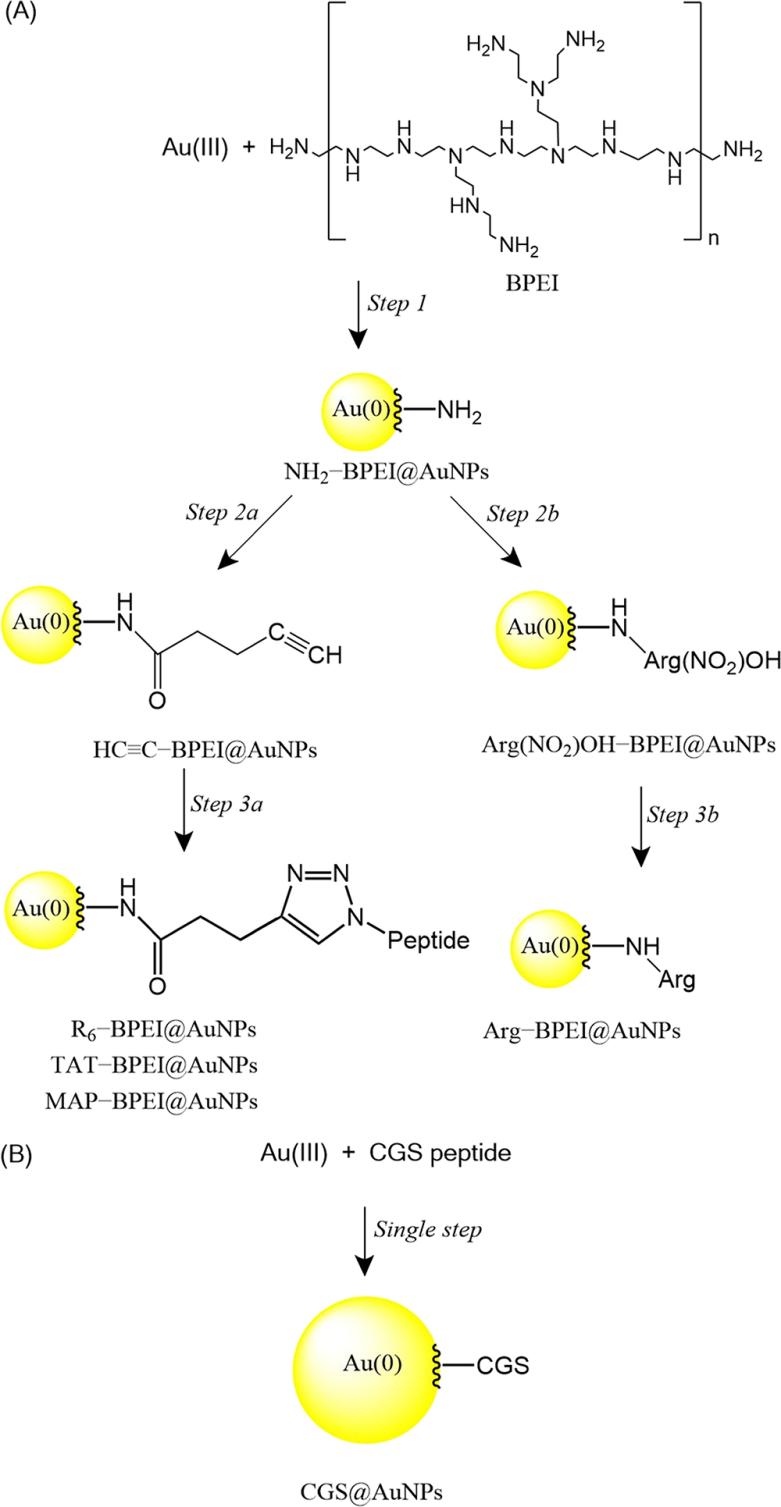
Envisioned Approaches to Synthesizing Peptide-Coated AuNPs: A Multistep General Platform Based on Selective Reactions (Peptide Coupling and Click Chemistry), Extensive to Virtually Any Peptide (A), and a Direct Single-Step, One-Pot, Two-Reactant Reaction without a Workup Protocol (B)

#### UV–vis Spectroscopy

UV–vis profiles were acquired using a Varian Cary 50 spectrometer and quartz cells with optical path length of 1.0 cm. The spectral resolution for wavelength scanning was 1.0 nm.

### Biological Assays

#### Cell Culture

HeLa cancer cells were cultured in DMEM supplemented with 10% fetal bovine serum and antibiotics (penicillin 10,000 units·mL^–1^ and streptomycin 10,000 μg·mL^–1^) to prevent bacterial contamination at 37 °C in a 5% CO_2_ atmosphere.

#### Cell Viability

HeLa cells at 10,000 cells/well were seeded in 96-well plates, grown for 24 h, and then incubated with AuNPs in fresh DMEM to a final volume of 100 μL. The cells were incubated with AuNPs for 24 h at 37 °C and 5% CO_2_, and then washed with fresh phosphate-buffered saline (PBS). The MTT reagent solution (50 μL at 0.3 mg·mL^–1^) was added to each well and incubated for 4 h under the same atmospheric conditions. The mitochondria convert the MTT reagent to formazan crystals. The formazan crystals were then dissolved in 150 μL of DMSO, and the absorbance at λ = 570 nm was measured using a Synergy microplate reader (untreated cells were used as a control to calculate cell viability %). Positive cell viability control (C+, 100% cell viability) consists of cells incubated with only the medium and no AuNPs under the same conditions. The negative cell viability control (C-, 0% cell viability) consists of wells filled with DMSO. All samples were prepared in at least triplicate and cell viability was then calculated as

$$CV{\%}_{spl}=100\times \frac{\left({\mathrm{A̅}}_{spl}-{\mathrm{A̅}}_{C-}\right)}{\left({\mathrm{A̅}}_{C+}-{\mathrm{A̅}}_{C-}\right)}$$ 4

where CV%_spl_ is the sample cell viability percentage, A̅_spl_ is the sample absorbance average, A̅_C‑_ is the negative control (0%) absorbance average, and A̅_C+_ is the positive control (100%) absorbance average.

The standard deviation of each % sample cell viability was calculated based on the propagation of uncertainty of simple functions as

$$\sigma_{CV\%spl}≅CV{\%}_{spl}\times \sqrt{{\left(\frac{\sqrt{{\sigma_{Aspl}}^{2}+{\sigma_{AC-}}^{2}}}{{\mathrm{A̅}}_{spl}-{\mathrm{A̅}}_{C-}}\right)}^{2}+{\left(\frac{\sqrt{{\sigma_{AC+}}^{2}+{\sigma_{AC-}}^{2}}}{{\mathrm{A̅}}_{C+}-{\mathrm{A̅}}_{C-}}\right)}^{2}}$$ 5

where σ _CV% spl_ is the standard deviation of the sample cell viability percentage, σ_Aspl_ is the standard deviation of the sample absorbance, σ_AC‑_ is the standard deviation of the negative control absorbance, and σ_AC+_ is the standard deviation of the positive control absorbance.

#### Cellular Uptake

HeLa cells at 50,000 cells/well were cultured in 48-well plates for 24 h. The culture medium was then removed, and the cells were washed with phosphate buffer solution and supplemented with free glucose DMEM culture medium. Afterward, the culture medium was replaced by 190 μL of free glucose DMEM and 10 μL of gold nanoparticles. The final concentration of AuNPs was kept to 50 μg·L^–1^ (0.05 ppm). The incubation was performed at 37 °C during different times (4, 8, and 24 h). After the incubation time, supernatants and cells on the cell-plates were dried in an incubator at 40 °C for 3 days. In the sequence, cells and supernatants were digested with 100 μL of *aqua regia* (HCl ∼ 36%: HNO_3_ ∼ 65% 3:1 *v*/*v*) for 30 min under stirring, and the resulting solution was diluted with 400 μL of ultrapure water. Total Au concentration was determined by an inductively coupled plasma mass spectrometer (ICP-MS Agilent 7900, Hachioji, Japan). The operational conditions of the apparatus were based on previous measurements.^26^ To minimize Au memory effects, two washing solutions (5% *v*/*v* aqua regia and ultrapure water) were used between each analysis. The measurements were performed in triplicate, and Au concentration values are given as mean ± standard deviation (n = 3).

### Statistical Analysis

The statistical analyses were conducted using the one-way analysis of variance (ANOVA) followed by the Tukey post hoc range test (p-value of 0.05 or less was considered to be statistically different). Population variations were not significantly different at 0.05 level as evaluated by Levene’s test for homogeneity of variances.

## Results and Discussion

### Synthesis and Characterization of Polymer- and Peptide-Coated AuNPs

The short, sequenced, and positively charged peptides listed in Table 1 were selected for this study because they belong to the so-called class of CPPs, which can translocate across lipid membranes, thus allowing for the intracellular delivery of cargo payloads without compromising the integrity of living cells.^27^ All these peptides are rich in arginine residues that are known to be effective in this regard.^28^ The synthesis of the six-arginine sequence was therefore inspired by such an experimental and proven fact, as well as the variant of the WQWRR sequence, known to show antimicrobial properties,^29^ which can also mediate the direct synthesis of the metallic nanoparticles as discussed hereafter. The main features of the CPPs are summarized in Table 1.

**Table 1:** Peptides Used in the Preparation of Peptide-Decorated AuNPs

**Peptide**	**Nomenclature**	**Function**	**Sequence**	**Molar Mass (g mol** ^**–1**^ **)**
R_6_	Six arginines	Possible cell-penetrating ability	N^–^=N^+^=N–CH_2_OO–GGGRRRRRR	1209
MAP	Model amphipathic peptide	Well-known cell-penetrating peptide	N^–^=N^+^=N––CH_2_OO– KLALKLALKALKAAL	2131
TAT	Trans-acting activator of transcription	Well-known peptide that can facilitate penetration into any cell type	N^–^=N^+^=N–CH_2_OO– GGGRKKKRRQRRR	1592
CGS	Lactoferricin-based WQWRR sequence extended with CGS residues	Antimicrobial activity and cell-penetrating sequence properties	CGSGWQWRR	1135

The three-step synthesis strategy developed in this study for preparing peptide-coated AuNPs is illustrated in Scheme 1A. First, a direct one-pot two-reactant synthesis of BPEI-capped gold colloids was carried out in DMF, with BPEI acting simultaneously as reducing and capping agent (step 1). The reaction characteristics were typical of AuNPs nucleation and growth, with color development as a function of time. The resulting NH_2_–BPEI@AuNPs in DMF were studied by imaging and scattering techniques. The experimental conditions used in this study consistently produced well-defined and uniformly distributed spherical particles with a mean diameter of 5.2 nm according to TEM micrographs and particle image analysis (see Figure 1). This characterization has also been complemented using UV–vis spectroscopy and dynamic light scattering, and the data are also provided in Figure 1.

**Figure 1:**
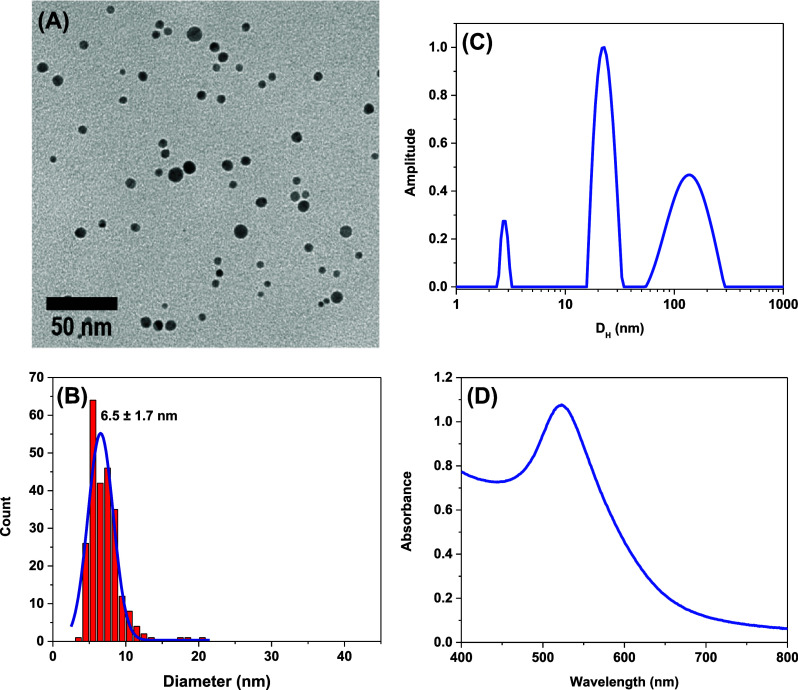
TEM micrograph (A) along with corresponding size distribution histogram (B), distribution of hydrodynamic diameters (C), and UV–vis spectrum (D) for NH_2_–BPEI@AuNPs synthesized in DMF.

Subsequently, amino groups at the surface of these particles reacted with 4-pentynoic acid to obtain alkyne-functionalized derivatives (step 2a). Peptide-coated AuNPs were finally prepared by Cu­(I)-catalyzed azide–alkyne cycloaddition (step 3a). The click reaction is efficient and fast, with a high degree of selectivity and stability, allowing us to use mild reaction conditions and aqueous media.^30,31^ The protocol previously devised, performed, and optimized by a member of our team and already described elsewhere was also applied in this study.^22^

The successful attachment of CPP at the particle surfaces was confirmed by surface analysis. The surface chemical composition of gold nanoparticles coated NH_2_–BPEI, HC≡C–BPEI, and TAT-BPEI layers was probed via X-ray photoelectron spectroscopy (XPS) technique. The high-resolution XPS spectra taken in the C 1s region of the nanoparticles (Figure 2) revealed the presence of dominant C–C and C–H contributions at 285.0 eV, along with a clear peak of C–N from the imine groups at 286.2 eV. In the spectra of NH_2_–BPEI, one can observe minor contributions at about 288.0 and 289.0 eV, most probably originating from amide and carboxyl defects present on the polymer chains. The amidation of the free amine groups of the BPEI chains leads to an obvious increase in the amide C­(=O)–NH contributions at 287.9 eV and a rise in the overall amount of oxygen species (Table 2). The incorporation of the TAT peptide in the PEI corona leads to further increase of the signals at 288.9 eV stemming from the presence of guanidino N–C­(=N)–NH and carboxy C­(=O)–O groups of the TAT peptide similarly as in the case of the reference spectra of free TAT peptide (Figure 2). Alongside the incorporation of the peptides leads to further increase of the oxygen signals as a result of the presence of the amide and carboxyl groups.

**Figure 2:**
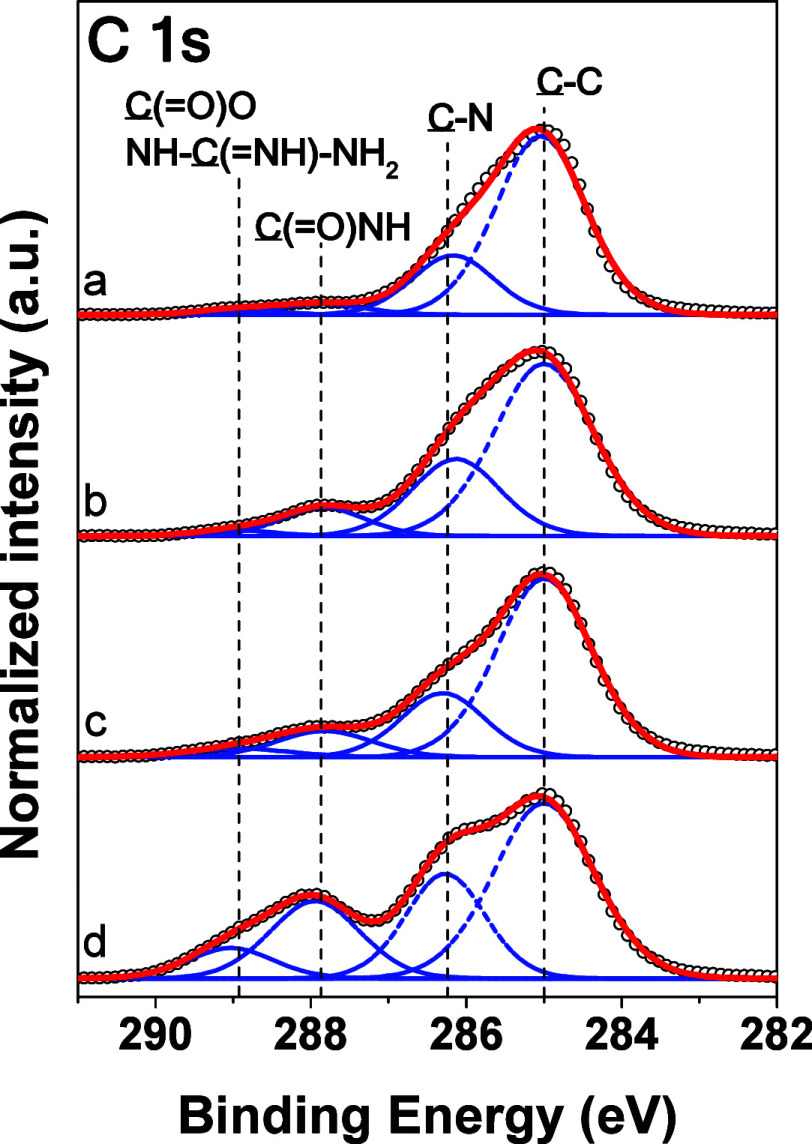
High resolution XPS spectra taken in the C 1s region for gold nanoparticles baring NH_2_–BPEI (a), HC≡C–BPEI (b) and TAT–BPEI (c) layers and free TAT peptide (d). (Black symbols: measured data; red curve: fitted data; blue: individual contributions of functional groups).

**Table 2:** Surface Composition of Gold Nanoparticles Bearing NH_2_–BPEI, HC≡C–BPEI, and TAT–BPEI Layers and Reference Free TAT Peptide as Determined from XPS Measurements

			**NH**_**2**_ **–BPEI**	**HC≡C–BPEI**	**TAT–BPEI**	**free TAT**
**Region**	**Chemical species**	**Position (eV)**	**atomic %**			
Au 4f	Au	84.2 ± 0.2	0.9 ± 0.1	1.2 ± 0.2	1.4 ± 0.1	-
Cl 2p	Cl ^–^	197.5 ± 0.2	0.4 ± 0.1	-^a^	-	-
C 1s	C–C, C–H	285	53.8 ± 1.7	46.8 ± 4.4	45.0 ± 3.7	27.4 ± 2.2
	C–N	286.2 ± 0.1	18.0 ± 1.6	19.1 ± 5.3	18.1 ± 6.3	12.4 ± 0.4
	C=O–NH	287.9 ± 0.1	3.9 ± 0.3	6.2 ± 0.7	6.4 ± 0.9	10.6 ± 0.7
	N–C(=N)–NH, C(=O)–O	288.9 ± 0.2	1.3 ± 0.2	1.2 ± 0.5	2.4 ± 0.3	3.7 ± 0.2
N 1s	N–C	399.8 ± 0.2	11.4 ± 0.5	11.2 ± 1.4	9.8 ± 0.5	22.4 ± 2.1
	N ^+^H	401.7 ± 0.2	2.8 ± 0.2	1.5 ± 0.2	1.2 ± 0.1	1.3 ± 0.1
O 1s	O–C, O=C	531.9 ± 0.2	7.6 ± 0.2	12.8 ± 0.6	15.9 ± 1.7	22.4 ± 0.4

a Below the detection limits of the XPS measurement, i.e., < 0.1 atomic %.

Considering that XPS has unmatched detection limits <0.1 atomic % and probes into a depth of a few nanometers only, we can reliably conclude from these results that peptides are present at the surface of the nanoparticles. Quantification of surface coverage is not appropriate in the context of this study because the surfaces are already rich in chemical elements present in the peptide sequences, namely carbon and nitrogen originating from BPEI. Consequently, deconvolution of the overlapping chemical environments cannot be reliably extended beyond a qualitative analysis. Additionally, the aggregated state of the particles in the dry samples may further complicate quantitative analysis. For these reasons, we chose to limit conclusions drawn from XPS to a qualitative proof of the presence of peptides on the nanoparticle surface. This is also robustly suggested by the biological studies and respective reported differences (discussed hereafter). Accordingly, these results enabled us to conclude that each step of surface modification was performed successfully, in line with previous findings.^22^

A different route was chosen for the synthesis of arginine-decorated particles. In this latter case, BPEI-decorated AuNPs reacted with activated Fmoc-Arg­(NO_2_)–OH (step 2b), followed by removal of the NO_2_ protecting group (step 3b). Each reaction step is described in detail in the experimental section.

The platform represented in Scheme 1, panel A, is demonstrated for positively charged CPPs. The use of a negatively charged peptide to functionalize positively charged particles may indeed introduce challenges similar to those encountered in polyelectrolyte complex systems. It is also true, however, that membrane-penetrating peptides investigated to date are predominantly positively charged, adhesive, or zwitterionic. This represents a likely limitation of the platform described herein, which remains to be evaluated.

An exception to this synthetic route is the CGS@AuNPs system, which was prepared directly in aqueous media by reacting CGS peptide (1.0 mg·mL^–1^; 1.5 mL) with HAuCl_4_ (1.0 mg·mL^–1^; 1.5 mL), as shown in Scheme 1B. In this case, the peptide acts simultaneously as both a reducing and a stabilizing agent, in accordance with previously reported reaction pathways.^15,16^ Although the other peptide sequences employed in this study also contain nitrogen-based chemical functionalities such as amines, amides, and amino acids, either within the backbone or as pendant groups, and are therefore capable of reducing auric species as previously reported, they do not confer colloidal stability to the resulting products. This behavior is attributed to the hydrophobic–hydrophilic balance of the amino acid residues, which in the case of the CGS peptide is more favorable for functioning as a capping agent, thereby providing effective steric and electrostatic stabilization.

Following such a route, a set of functional gold colloids can be prepared through a consistent synthetic strategy that facilitates the tailoring of gold colloid surface chemistry with ease. We believe this represents a clear step forward in the field of gold nanoparticle functionalization for theranostic applications, as the same protocol can be used to isolate such variables.

All the capped nanoparticles were fully characterized using TEM, DLS, ELS, and UV–vis spectroscopy. Integrated multiple characterization results are provided in Figure 3 with quantitative assessment summarized in Table 3. In the present study, TEM and DLS are techniques that aim to probe different aspects of the nanoparticles, being complementary to each other. Indeed, with the widespread use of DLS and TEM for nanoparticle analysis, it is crucial to consider the fundamental aspects and differences in the results provided by both, as discussed by Filippov et al.*.* ^32^ Data obtained by TEM reveal the size of the metallic core only. Apart from the CGS@AuNPs system, the results for the *D*_TEM_ indicated the same value as expected because all the functionalized particles originated from the same source (NH_2_–BPEI@AuNPs starting reactant, see step 1 in Scheme 1). Much larger gold cores were evidenced for CGS@AuNPs (see Figure 3). In such a case, the nucleation and growth are mediated in the aqueous media (instead of DMF), and the reducing agent has a disparate chemical nature (CGS peptide sequence instead of BPEI chains).

**Table 3:** Hydrodynamic Diameter and Polydispersity Index Determined by DLS (*D*_H_ and *PdI*), Diameters from Particle Image Analysis by TEM (*D*_TEM_), Zeta Potential (ζ), and Wavelength at Maximum Absorption (λ_max_) for AuNPs Synthesized Using Various Stabilizers/Functional Coatings According to Labels

**Entry**	**D**_**H**_ **(nm)**	**PdI**	**ζ (mV)**	**D**_**TEM**_ **(nm)**	**λ**_**max**_ **(nm)**
NH2–BPEI@AuNPs	22	0.35	+ 42	6.5	528
MAP–BPEI@AuNPs	31	0.53	+ 44	5.5	530
TAT–BPEI@AuNPs	22	0.56	+ 28	5.2	529
R_6_–BPEI@AuNPs	23	0.57	+ 25	5.5	529
Arg–BPEI@AuNPs	32^a^	-	+ 44	6.3	527
CGS@AuNPs	62	0.35	+ 35	30.6	533

a Estimated using the inverse Laplace transformation method to analyze the autocorrelation function.

**Figure 3:**
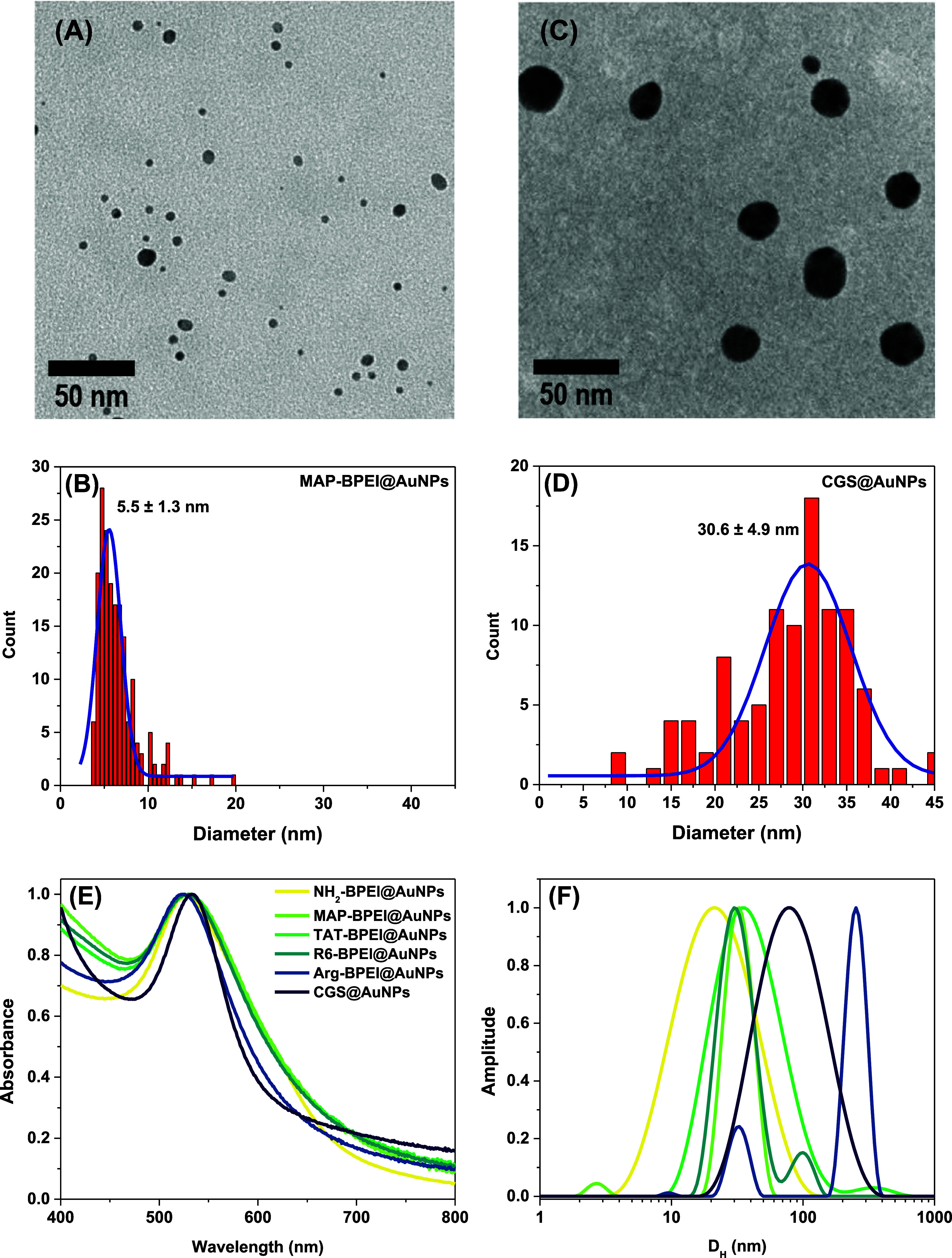
TEM micrograph and corresponding size distribution histogram for MAP–BPEI@AuNPs (A, B) and CGS@AuNPs (C, D), along with integrated multiple characterization UV–vis spectra (E) and light scattering size distributions (F).

The *D*_*H*_ values reflected the intensity-averaged distribution of relaxation times, which is the fundamental distribution generated by fluctuations of the scattered light intensity that effectively carry the dynamics of scattering particles. Therefore, it is the most pristine signal to be interpreted during data analysis, as it conveys information on molecular interactions, repulsions, and other relevant phenomena. The values shown in Table 3 for the first five entries indicate that MAP, TAT, and R_6_ peptide sequences behave similarly in terms of chain conformation at the surface of the gold core, leading to similar particle solution dynamics, as evidenced by their comparable *D*_*H*_ values. The Arg–BPEI@AuNPs particles were found to form dynamic aggregates in solution through interparticle interactions (scattering objects with large hydrodynamic dimensions); therefore, we report in Table 3 the estimated value of *D*_H_ provided by using the CONTIN method (inverse Laplace transformation) for analyzing the autocorrelation function. The term dynamic aggregate refers to reversible, transient assemblies of particles or macromolecules that continuously form, dissociate, and reorganize due to thermal motion and intermolecular interactions. Importantly, there are no chemical bonds between these particles (no chemical cross-linking). Because of this dynamic nature, the broad distributions do not compromise quantitative comparison of uptake data or cytotoxicity outcomes. In the case of CGS@AuNPs, *D*_H_ and *D*_TEM_ differ significantly less, indicating that the particles follow essentially the same behavior as MAP, TAT, and R_6_ analogs. Overall, the polydispersity indexes indicate broad size distribution. In this regard, it is worth mentioning that the size of the metallic core determined by TEM is uniform (5.8 ± 0.6 nm), and that the broad size distribution is found by DLS due to the presence of dynamic aggregates in solution (as discussed above), which are in fact expected in the case of polyelectrolytes. Nevertheless, postsynthesis purification such as by using field-flow fractionation (FFF) or size-exclusion chromatography, as well as changes in the electrolyte composition, may help in preventing the formation of dynamic aggregates. Concerning the size of the metallic core, control is achievable when using BPEI by adjusting the composition of the aqueous reaction environment.^15^ The chemical nature of the polymer chain also has a pronounced effect on nucleation and growth rates. For instance, reducing agents with lower electron availability, such as poly­(2-methyl-2-oxazoline) are suitable for synthesizing spherical AuNPs with larger size,^16^ but the polymer composition would have to the designed to feature amino groups in order to fit to the herein envisioned synthetic approach of which Step 2a in Scheme 1 (synthesis of alkyne-functionalized gold colloids) is key.

The zeta potential values reported in Table 3 are consistent with the colloidal stability observed by UV–vis spectroscopy over time. The stability of this type of colloid arises from a complex equilibrium of forces and a synergic effect involving both steric and electrostatic contributions, which are clearly present in the peptide-functionalized nanoparticles developed in this study. All nanoconstructs are positively charged, consistent with the presence of protonated amine groups originating either from BPEI chains or from the peptide sequences. The values close or above +30 mV point to a prominent electrostatic stabilization effect. Therefore, aggregation is prevented even at relatively high concentrations, due to electrostatic repulsion arising from surface charge–induced interactions between particles. Indeed, the particle systems listed in Table 3 remain stable under high ionic strength conditions with no significant spectral changes observed up to a concentration of 5 ppm as evidenced by UV–vis measurements. Furthermore, the data underlines typical and well-defined SPR band observed for all particulate systems, with λ_max_ centered at approximately 528 nm, indicating the presence of spherical or quasi-spherical gold nanoparticles with diameters ranging from 6 to 30 nm.^33^

### Evaluation of Cell Cytotoxicity

As stated by Fratoddi et al.,^34^ the evaluation of cell cytotoxicity of gold colloids is a puzzle that calls for a more comprehensible scenario. We discussed this issue recently^16^ and consider here as well the same integrative approach to data interpretation that takes into account the size, number, and surface chemistry of particles. Figure 4 shows the dose-dependent viability of HeLa cells incubated in the presence of AuNPs with different coatings (polymer and peptide sequences) as assessed by the MTT assay. The dashed line in Figure 4 indicates the 70% cell viability threshold, which is commonly used to assess cytotoxic effects according to ISO 10993–5:2009, an international standard for evaluating *in vitro* cytotoxicity. According to this standard, a reduction in cell viability of more than 30% is indicative of cytotoxic effects, whereas cell viability ≥ 70% is considered indicative of noncytotoxicity. The results shown in Figure 4 demonstrate that cell viability remains above 70% up to a concentration of 1.0 ppm, regardless of the chemical nature of the nanoparticle surface. Therefore, under the experimental conditions employed, the nanoparticles synthesized in this study do not induce cytotoxic effects in HeLa cells.

**Figure 4:**
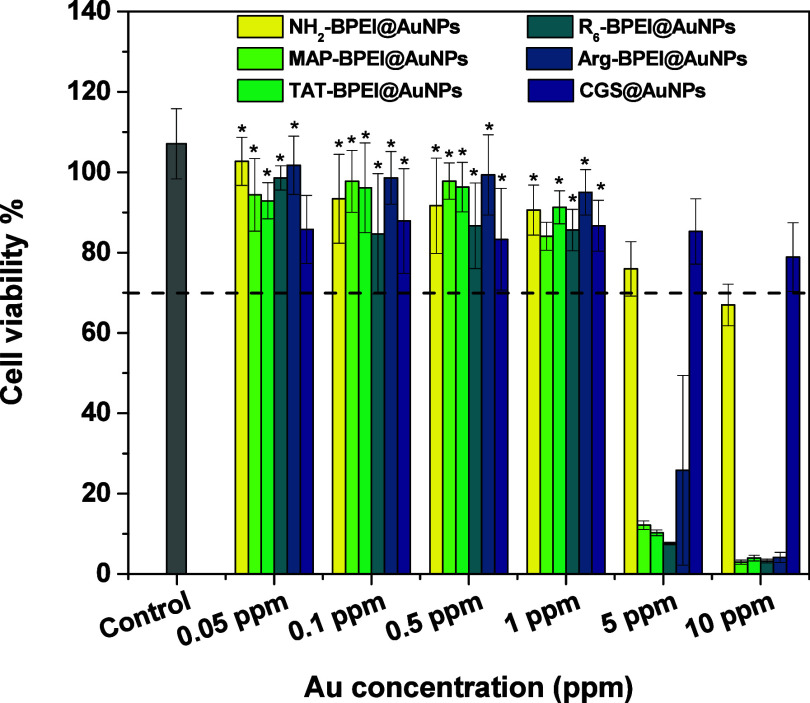
Dose-dependent viability of HeLa cells incubated with AuNPs capped with different coatings according to the legend. The control refers to untreated cells (the results are expressed as mean ± standard deviation). *Not statistically different compared to the control according to statistical ANOVA and Tukey analysis at *p* = 0.05 level.

It is very interesting to recall that all but CGS@AuNPs systems were built from the same NH_2_–BPEI@AuNPs precursor (see step 1 in Scheme 1). Therefore, the size of the metallic core is identical across the set of samples derived from the NH_2_–BPEI@AuNPs precursor. Consequently, the same number of particles is present in all these cases. The CGS@AuNPs system is the only exception. NH_2_–BPEI@AuNPs is clearly less toxic than particles featuring CPPs at the outermost surface; cells remain viable up to 5.0 ppm for NH_2_–BPEI@AuNPs rather than 1.0 ppm for MAP–BPEI@AuNPs, TAT–BPEI@AuNPs, R_6_–BPEI@AuNPs and Arg–BPEI@AuNPs. BPEI chains consist of primary, secondary, and tertiary amines with different p*K*_a_ values. However, the precise values cannot be easily assigned due to the effect of the surrounding environment, molecular interactions, and polyelectrolyte behavior. The chemical substitutions investigated herein primarily convert primary to secondary amines. This chemical reaction alters the cationic charge density of the polycations, thereby modifying the interaction of the polymeric materials with cell membranes and, in all cases, enhancing cell damage. The induced cytotoxicity is indeed clearly visible in the CPP-functionalized particles. The cytotoxicity of catiomers^35,36^ is usually associated with electrostatic interactions with a variety of negatively charged lipids present in several cell organelles, promoting, usually, cell apoptosis. The actual mechanism of cell death occurring in these systems is being probed by flow cytometry analysis with dual fluorescent staining (propidium iodide (PI) and annexin-V) to distinguish necrosis from apoptosis (annexin V-positive/PI-negative staining implies apoptosis whereas PI-positive staining suggests necrosis as the main mechanism).

The low levels of cell cytotoxicity found for CGS@AuNPs are due to the small number of particles. At the same Au concentration, the total amount of Au atoms is distributed over a much smaller number of particles because the larger CGS@AuNPs contain more material per particle. Consequently, the number of particles in CGS@AuNPs systems is approximately 150-fold lower than that in CPP–BPEI@AuNPs derived from the NH_2_–BPEI@AuNPs precursor. For the sake of comparison, the number of particles calculated using the approach described by Fratoddi et al.^34^ at 1.0 ppm is 3.32 × 10^9^ particles·mL^-1^ for CGS@AuNPs and 5.07 × 10^11^ particles.mL^-1^ for CPP–BPEI@AuNPs, considering an average particle radius of 15.5 and 2.9 nm, respectively. Cell death is closely related to the quantity of nanoparticles that accumulate within the cellular environment, triggering deadly responses, particularly those affecting equilibria involving reactive oxygen species.^16,37,38^ It is of relevance noting that residual copper from the cycloaddition reaction as source of toxicity can be disregarded. Particles were double washed with 1% EDTA after the copper-catalyzed click reaction. The efficiency of such a protocol was confirmed in previous work by the absence of radioactivity originating from ^64^Cu.^22^ Therefore, since AuNPs themselves are known to be nontoxic, cell damage is due to the coating material which features positive charges known to be effective in disrupting cell membranes. Although cytotoxicity depends on the cell line and structural features of the nanomaterial (surface, shape, surface charge), cell damage has been evidenced for BPEI@AuNPs in concentrations higher than 10 ppm,^39,40^ therefore in agreement with the results of this study.

### Evaluation of Cellular Uptake

The ability of polymer or peptide-coated AuNPs to translocate across the lipidic cellular membrane was evaluated after distinct exposure times based on established protocols reported elsewhere.^9,26^ Cultured HeLa cells were incubated in the presence of particles at 37 °C and digested for subsequent quantification of the internalized amounts of Au by ICP-MS. The final concentration of AuNPs was kept at 0.05 ppm for cellular uptake as described in detail in the experimental section. This value has been chosen to avoid artificial effects on the biological performance linked to nanoparticle aggregation. We observed that particles remain stable in high ionic strength environment with no significant spectral differences up to a concentration of 5 ppm (not shown for brevity) thanks to electrostatic stabilization provided by the determined positive charges at the surface.

The results are presented in Figure 5 as the percentage of gold found inside HeLa cells for various incubation periods.

**Figure 5:**
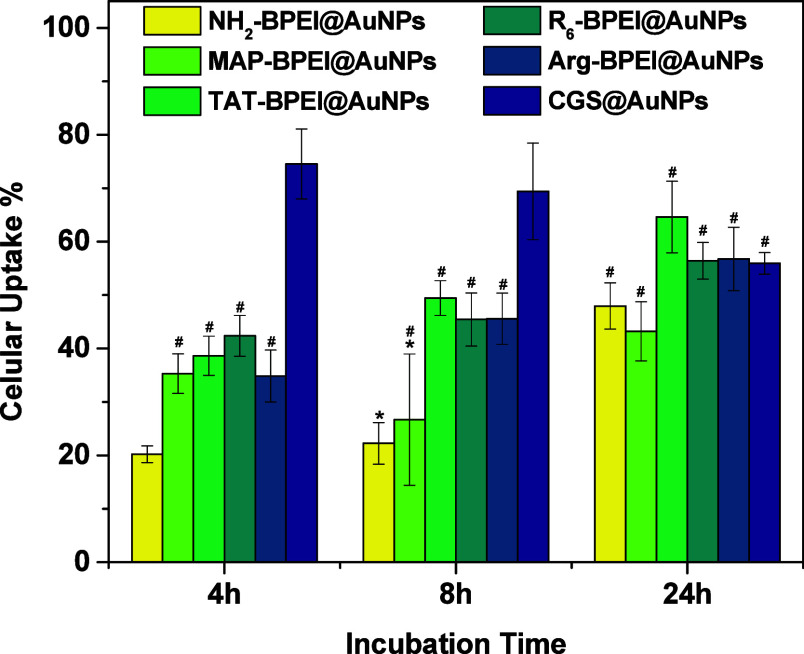
HeLa cellular uptake after different incubation times of AuNPs capped with different coatings according to the legend (data are expressed as mean ± standard deviation with *n* = 3). *^,#^ Not statistically different compared to each other at *p* = 0.05 level as concluded from statistical ANOVA and Tukey analysis.

At 0.05 ppm mass-based Au concentration, the corresponding particle-number-normalized concentration is ca. 2.53 × 10^10^ particles·mL^-1^ for all systems derived from the NH_2_–BPEI@AuNPs precursor (i.e.: NH_2_–BPEI@AuNPs and CPP–BPEI@AuNPs). Therefore, comparison among these systems is straightforward, as the normalization involves a constant factor. The ligand density is normalized across these samples. The only exception is CGS@AuNPs, which are present at an approximately 150-fold lower concentration of 1.66 × 10^8^ particles·mL^-1^.

Observing the data obtained for the NH_2_–BPEI@AuNPs system as a reference for peptide-free nanoobjects, the experimental data put forward, at a first glance, evidence for the effect of surface peptide chemistry on the cellular uptake behavior, particularly on the rate of the overall process. NH_2_–BPEI@AuNPs were slowly uptaken as a function of time, from approximately 20% during 4 h incubation time. The process is clearly faster when CPPs were attached to the surface of particles (TAT–BPEI@AuNPs, MAP–BPEI@AuNPs, and R_6_–BPEI@AuNPs), as internalization quickly reached ca. 40% after the same incubation time, and continued to gently increase above the mark registered in the absence of CPPs. These findings reveal the pure effect of CPP functionalization as particle size is kept constant, and thus the size-dependent endocytosis^41^ variable is controlled.

The highest values of cellular uptake have been determined for CGS@AuNPs. Under the experimental conditions used in this part of the study, with Au mass concentration kept at a constant value in all cases, the number of CGS@AuNPs is ca. 150-fold smaller than that of CPP–BPEI@AuNPs analogs because the former exist as large objects. Even in lower abundance, CGS@AuNPs do translocate into the cellular environment preferably.

Based on the comprehensive review by Hoshyar et al.*,* ^42^ this behavior may be linked to a size-dependent, membrane-wrapping driven uptake of ligand-coated nanoparticles, since nanoparticles ranging from 30 to 60 nm are known to mediate the membrane-wrapping process more effectively. In addition, Yuan et al.^43^ discovered that a limited number of ligands must bind in proximity on the surface to drive the membrane-wrapping process with an enthalpic limit around 30 nm and an entropic penalty appearing above 60 nm. Correlating the characteristics of the CGS@AuNPs with these studies, it is plausible that the high surface concentration of CPPs on CGS@AuNPs contributes to enhanced cytosolic uptake. Conversely, CPP–BPEI@AuNPs, which present a lower surface density of peptides, are more likely to be internalized predominantly via endocytic pathways rather than through direct cytosolic translocation.^27^

It is quite remarkable that the best outcomes were achieved with the simplest of the strategies: a short CPP sequence that successfully mediates the formation of stable gold colloids in one-pot, two-reactant, no-workup reactions, with an overall size within the optimal range for size-dependent internalization.

## Conclusions

From the results of this study, two main conclusions were drawn. First and foremost, the devised platform for anchoring virtually any peptide at the surface of gold colloids was effective. This claim is supported by data showing higher cellular uptake for all CPP–BPEI@AuNPs than for NH_2_–BPEI@AuNPs precursor, while all other parameters were kept under control. A possible limitation of this methodology may arise from the use of negatively charged peptides to functionalize positively charged particles, a scenario that may pose challenges as typically seen in polyelectrolyte complexes of macromolecules. However, it is true that membrane-penetrating peptides tested so far in our group and others are positively charged, adhesive or zwitterionic. Second, smart synthetic approaches for strategically modifying well-known CPP sequences, such as WQWRR, to improve solubility properties can be envisioned to generate new peptide sequences, such as CGSWQWRR, capable of doing it all: mediating the formation and sterically stabilizing gold nanoparticles within the optimal size range for inducing translocation into the cellular environment.

The findings of this study reduce the gap between synthesis and practical applications of nanoparticles that require cellular internalization by bringing effective simplicity to nanoparticle manufacturing.
